# Genomic insights into a robust gamma-aminobutyric acid-producer *Lactobacillus brevis* CD0817

**DOI:** 10.1186/s13568-019-0799-0

**Published:** 2019-05-24

**Authors:** Dandan Gao, Kunpeng Chang, Gongtao Ding, Hongjing Wu, Yuanhong Chen, Mengya Jia, Xiaohua Liu, Shuixing Wang, Yuyuan Jin, Hao Pan, Haixing Li

**Affiliations:** 1 Biomedical Research Center, College of Life Sciences and Engineering, Northwest Minzu University, Lanzhou, 730030 Gansu People’s Republic of China; 2 State Key Laboratory of Food Science and Technology, Nanchang University, Nanchang, 330047 Jiangxi People’s Republic of China; 3 Sino-German Joint Research Institute, Nanchang University, Nanchang, 330047 Jiangxi People’s Republic of China; 4 Nanchang University College of Science and Technology, Nanchang, 330029 Jiangxi People’s Republic of China

**Keywords:** *Lactobacillus brevis* CD0817, Complete genome sequence, Comparative genomics analysis, Gamma-aminobutyric acid, Glutamic acid decarboxylase

## Abstract

*Lactobacillus brevis* CD0817, a strain isolated from a healthy adult gut, was currently the most efficient lactic acid bacterial cell factory for gamma-aminobutyric acid. In this study, the complete genome sequence of CD0817 was determined and compared with some related *L. brevis* genomes. The CD0817 genome consists of one 2,990,570-bp chromosome and four plasmids. The comparative genomic and phylogenetic analysis revealed that *L. brevis* CD0817 was not very conserved with low GABA-producing *L. brevis* strains. A significant divergence was that CD0817 harbors only the *gadCA* operon whereas the low GABA-producing *L. brevis* strains contain the operon and *gadB*. The *gadB* seemed to only marginally contribute to the accumulation of GABA. The high GABA production ability of CD0817 may be associated with its extraordinary genome.

**Electronic supplementary material:**

The online version of this article (10.1186/s13568-019-0799-0) contains supplementary material, which is available to authorized users.

## Introduction

Gamma-aminobutyric acid (GABA), a four-carbon non-protein amino acid diffusely distributed in nature, is the major inhibitory neurotransmitter in the mammalian central nervous system (Li and Cao 2010). GABA has been widely applied in pharmaceutical, food and feed industries, due to its important physiological functions, such as anti-anxiety (Wong et al. 2003), hypotensive (Inoue et al. 2003), and diuretic effects (Nikmaram et al. 2017). Over the past three decades, the bio-manufacture of GABA by using lactic acid bacteria (LAB) has been vigorously pursued due to the fact that LAB are generally regarded as safe (Li and Cao 2010). Numerous LAB strains, notably lactobacilli belonging to *L. brevis* (Wu et al. 2015), *L. plantarum* (Siragusa et al. 2007), *L. paracasei* (Komatsuzaki et al. 2005) and *L. buchneri* (Zhao et al. 2015) have been applied to biosynthesize GABA.

Glutamic acid decarboxylase (GAD) system (namely GABA synthesizer) comprising glutamate/GABA antiporter (encoded by *gadC*) and GAD enzyme (encoded by *gadA* or *gadB*) is responsible for the synthesis of GABA in microbial cells: glutamate is transported into a cell through the antiporter, subsequently decarboxylation occurs, finally the decarboxylated product is exported from the cell by the antiporter (Small and Waterman 1998). Of the three genes, *gadCA* form an operon while *gadB* is separate and far from the operon circa 1.7 Mb in a *L. brevis* genome (Li et al. 2013).

It is intriguing that low GABA-producing *L. brevis* strains possessing an identical GAD system exhibited entirely different GABA-synthesizing ability (Li and Cao 2010). It was therefore presumed that the formation of GABA may also be associated with cell physiological status essentially determined by genetic information. Data at genome level may help us to understand the causes regarding this discrepancy in GABA yield. However, of all the reported GABA-producing *L. brevis* strains, no more than the genome of *L. brevis* NPS-QW-145 is currently available (Wu et al. 2015; Wu and Shah 2015); on the other hand, only strain NPS-QW-145 has been clearly demonstrated to generate GABA among the several genome-sequenced *L. brevis* strains.

Recently, a strain *L. brevis* CD0817 with the highest known GABA production (252 g/L) among LAB strains was screened from the gut of a healthy adult (Chen et al. 2019). Herein, the complete genome sequence of CD0817 was reported and compared with some other completely sequenced *L. brevis* genomes; and the GAD system of CD0817 was highlighted. This work would enrich the genome database of GABA-producing LAB, and thus may help us to seek the reasons for the GABA yield difference then effectively elevate lactic acid bacterial GABA production by improving or regulating a strain.

## Materials and methods

### Bacterial strain, media and cultivation

*Lactobacillus brevis* CD0817 (= CCTCCM2018462) was isolated from a fecal sample of a healthy adult (Chen et al. 2019). The seed medium (pH 5.0) contained (g/L): glucose, 50; yeast extract, 25; monosodium l-glutamate, 28; manganese sulfate, 0.01; and Tween-80, 2. The fermentation medium was (g/L): glucose, 25; yeast extract, 25; l-glutamic acid, 515; manganese sulfate, 0.025; and Tween-80, 2. Glucose, l-glutamic acid, and the other components of the fermentation medium were separately autoclaved at 121 °C for 30 min and mixed together prior to inoculation. The *L. brevis* CD0817 cells were incubated in the seed medium at 32 °C and 100 rpm for 5–10 h till the absorbance at 600 nm reached 4.0–6.0 and then could be used as inoculum. Ten mL the seed was transferred into a 250-mL flask containing 100 mL the fermentation medium then statically incubated at 32 °C for 60 h. The GABA concentrations in the fermentation broths were determined by a previously described HPLC method (Li et al. 2009).

### Genome sequencing and assembly

Genomic DNA was extracted from CD0817 cells using TIANamp Bacteria DNA Kit (Tiangen Biotech, Beijing, China) according to the standard protocol as recommended by the manufacturer. Total DNA obtained was subjected to quality control by 1% agarose gel electrophoresis, and the final concentration was determined by Qubit 2.0 Fluorometer (Life Technology, USA). The genome was sequenced with MPS (massively parallel sequencing) Illumina technology. Three DNA libraries were constructed: one paired-end (PE) library with an insert size of 500 bp; two mate-pair (MP) libraries with insert sizes of 2 kb and 5 kb, respectively. The PE and MP libraries were sequenced using Illumina Miseq platform by PE250 strategy and Illumina HiSeq2500 platform by PE125 strategy, respectively. Library construction and sequencing were performed at Novogene Bioinformatics Technology Co., Ltd (Beijing, China). Quality control of both PE and MP reads was performed using in-house program. After this step, Illumina PCR adapter reads and low-quality reads were filtered. The filtered reads were de novo assembled by SOAPdenovo (Li et al. 2010; Luo et al. 2012) (http://soap.genomics.org.cn/soapdenovo.html) to generate scaffolds. All reads were used for further gap closure.

### Genome component prediction

A program of tRNAscan-SE (Lowe and Eddy 1997) was used to predict tRNA genes. rRNA genes were identified with rRNAmmer (Lagesen et al. 2007) and sRNAs were predicted by BLAST against Rfam (Nawrocki et al. 2015) database. Repetitive sequences were predicted using RepeatMasker (Saha et al. 2008) (http://www.repeatmasker.org/). Tandem repeats were analyzed using Tandem Repeat Finder (Benson 1999) (http://tandem.bu.edu/trf/trf.html). Genomic islands were predicted by IslandPath-DIOMB (Hsiao et al. 2003). PHASTER (Arndt et al. 2016) (http://phaster.ca/) and CRISPRFinder (Grissa et al. 2007) were used to predict prophages and clustered regularly interspaced short palindromic repeats (CRISPRs), respectively.

### Genome annotation

Protein-coding gene prediction was performed on CD0817 genome assembly by GeneMarkS (Besemer et al. 2001) (http://topaz.gatech.edu/) with integrated model combining the GeneMarkS generated (native) and Heuristic model parameters. A whole genome Blast search (E-value less than 1e−5, minimal alignment length percentage larger than 40%) (Altschul et al. 1990) was performed against the following 6 databases: COG (Clusters of Orthologous Groups) (Tatusov et al. 1997), GO (Gene Ontology) (Ashburner et al. 2000), KEGG (Kyoto Encyclopedia of Genes and Genomes) (Kanehisa et al. 2006), Swiss-Prot, NR (Non-Redundant Protein Database), and TrEMBL (Magrane and UniProt 2011). Genome overview was created by Circos (Krzywinski et al. 2009) to show annotation information.

### Comparative genomic and phylogenetic analyses

The genomic features and GABA yields of CD0817 and 27 reference lactobacilli strains used in this study are listed in Table 1. Core/Pan genes of CD0817 and the 15 completely sequenced *L. brevis* strains were clustered by the Cd-hit (Li and Godzik 2006) software with a threshold of 50% pairwise identity and 0.7 length difference cutoff in amino acid. Gene family was constructed with the protein-coding genes of CD0817 and the 15 *L. brevis* strains, using multi softwares: Blast (Altschul et al. 1990) was used to pairwise align all protein-coding genes and the redundancy was eliminated by Solar and gene family clustering treatment for the alignment results was carried out with Hcluster_sg software. The phylogenetic trees were respectively constructed for the GAD genes retrieved from the 28 lactobacilli strains and the 718 single-copy orthologous genes detected from the gene family analysis across 16 completely sequenced *L. brevis* strains by the TreeBeST (Nandi et al. 2010) using the method of PhyML with 1000 replications. Synteny analysis between CD0817 and NPS-QW-145 (Wu et al. 2015) was performed using MUMmer (Kurtz et al. 2004) and LASTZ (Chiaromonte et al. 2002) alignment tools.

**Table 1:** Genomic features and GABA yields of the lactobacilli strains used in this study

Lactobacilli strains	Size (Mb)	GC content (%)	No. of protein-coding gene	No. of plasmid	GenBank/RefSeq accession number	GABA yield (g/L)	References
*L. brevis* CD0817	3.10	50.35	2990	4	CP032931.1	252	This study
*L. brevis* ATCC 367	2.34	46.04	2180	2	NC_008497.1	NA	Makarova et al. (2006)
*L. brevis* KB290	2.59	45.57	2457	9	NC_020819.1	NA	Fukao et al. (2013)
*L. brevis* NPS-QW-145	2.55	45.80	2391	0	NZ_CP015398.1	25.83	Wu et al. (2015)
*L. brevis* TMW 1.2112	2.67	45.72	2338	5	NZ_CP016797.1	NA	Fraunhofer et al. (2018)
*L. brevis* TMW 1.2113	2.67	45.70	2321	4	NZ_CP019750.1	NA	NA
*L. brevis* TMW 1.2108	2.92	45.27	2738	8	NZ_CP019734.1	NA	NA
*L. brevis* TMW 1.2111	2.88	45.31	2508	6	NZ_CP019743.1	NA	NA
*L. brevis* 100D8	2.48	45.75	2355	3	NZ_CP015338.1	NA	NA
*L. brevis* SRCM101174	2.57	45.59	2474	5	NZ_CP021478.1	NA	NA
*L. brevis* SRCM101106	2.55	45.60	2421	4	NZ_CP021672.1	NA	NA
*L. brevis* BDGP6	2.79	45.60	2679	0	NZ_CP024635.1	NA	NA
*L. brevis* ZLB004	2.66	45.61	2420	5	NZ_CP021456.1	NA	NA
*L. brevis* LMT1-73	2.53	45.89	2347	2	NZ_CP033885.1	NA	NA
*L. brevis* NCTC13768	2.49	46.00	2356	0	NZ_LS483405.1	NA	NA
*L. brevis* BSO 464	2.72	45.46	2606	8	CP005977.1	NA	Bergsveinson et al. (2015)
*L. brevis* CGMCC 1306	NA	NA	NA	NA	NA	76.36	Fan et al. (2012)
*L. brevis* HYE1	NA	NA	NA	NA	NA	1.93	Lim et al. (2017)
*L. brevis* 877G	NA	NA	NA	NA	NA	1.91	Seo et al. (2013)
*L. brevis* IFO 12005	NA	NA	NA	NA	NA	1.05	Yokoyama et al. (2002)
*L. brevis* OPK-3	NA	NA	NA	NA	NA	2.02	Park and Oh (2007)
*L. paracasei* NFRI 7415	NA	NA	NA	NA	NA	31.14	Komatsuzaki et al. (2008)
*L. plantrarum* WCFS1	3.35	44.45	3063	3	NC_004567.2	NA	Kleerebezem et al. (2003)
*L. paraplantarum* L-ZS9	3.14	44.00	2835	0	NZ_CP013130.1	NA	Liu and Li (2016)
*L. reuteri* TD1	2.15	38.80	1890	0	NC_021872.1	NA	Leonard et al. (2014)
*L. fermentum* F-6	2.06	51.70	1874	0	NC_021235.1	NA	Sun et al. (2015b)
*L. buchneri* NRRL B-30929	2.59	44.21	2368	3	NC_015420.1	NA	Liu et al. (2011)
*L. oris* J-1	3.24	51.36	2868	2	CP014787.1	NA	NA

NA: not available; the CD0817 and 15 completely sequenced *Lactobacillus brevis* strains were selected for comparative genomics analysis; all lactobacilli strains listed in the table were selected for GAD analysis

### Nucleotide sequence accession number

The CD0817 complete genome sequence data has been deposited in the GenBank database under the accession numbers of CP032931.1 (chromosome), CP032932.1 (pCD0817-1), CP032933.1 (pCD0817-2), CP032934.1 (pCD0817-3), and CP032935.1 (pCD0817-4).

## Results

### General genomic features of *L. brevis* CD0817

**Fig. 1:**
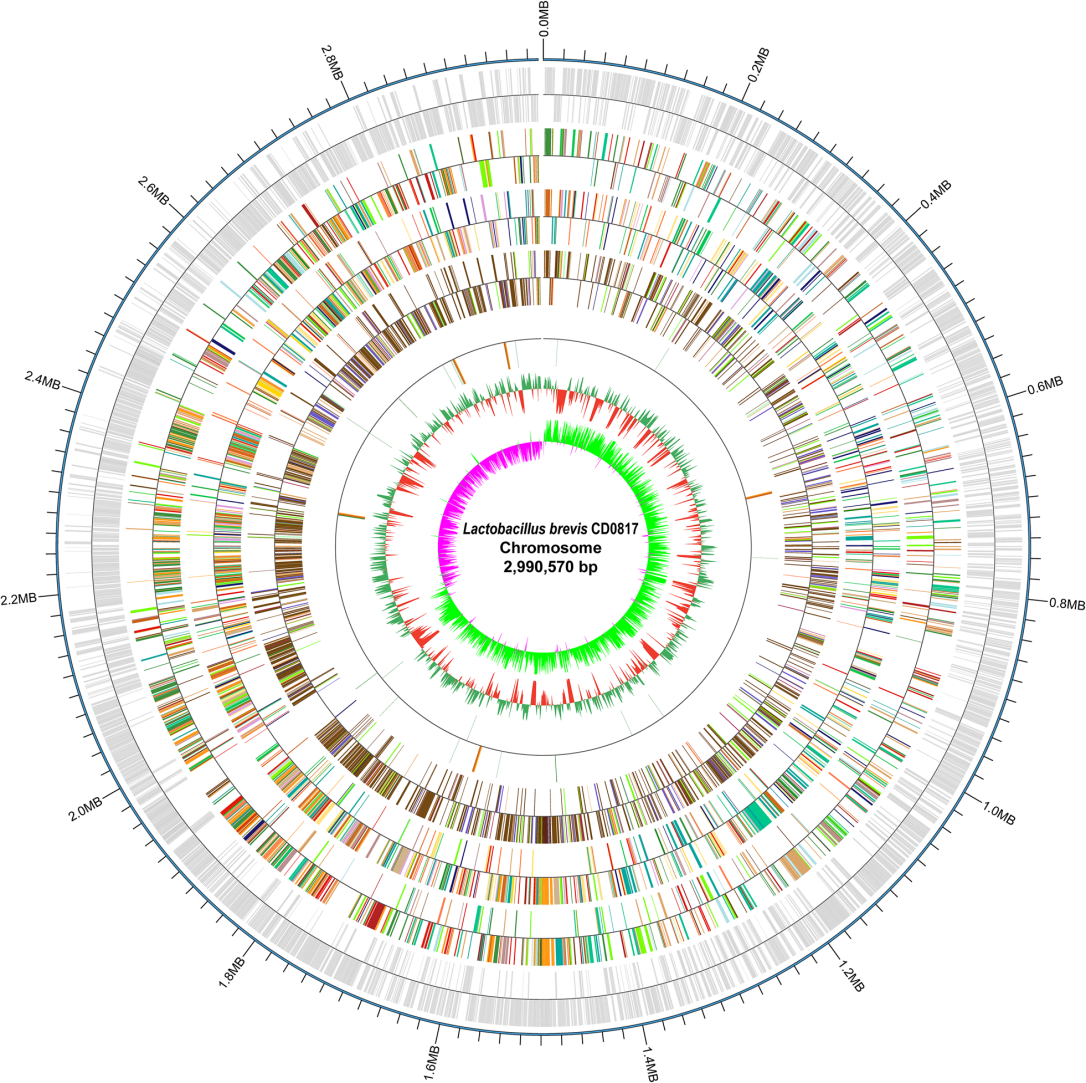
Graphical circular map of CD0817 chromosome. The 13 concentric circles represent the following (from outermost to innermost): circle 1, DNA base position; circles 2 and 3, protein-coding genes on forward and reverse strands; circles 4 and 5, COG functional classification of protein-coding genes on forward and reverse strands; circles 6 and 7, KEGG functional classification of protein-coding genes on forward and reverse strands; circles 8 and 9, GO functional classification of protein-coding genes on forward and reverse strands; circles 10 and 11, rRNA (5S, 16S and 23S), tRNA and sRNA genes on forward and reverse strands; circle 12, relative G + C content, green (outward) and red (inward) indicate higher and lower than average value of 50.63%, respectively; circle 13, GC skew ([G − C]/[G + C]), lime (outward) and magenta (inward) denote positive (representative of leading strand) and negative (representative of lagging strand) values, respectively

The principal features of CD0817 genome are visualized in Fig. 1. The genome comprises one 2,990,570-bp circular chromosome with an average GC content of 50.63% and four distinct plasmids designated as pCD0817-1 (37,310 bp), pCD0817-2 (30,761 bp), pCD0817-3 (26,298 bp), and pCD0817-4 (11,935 bp) with mean GC contents of 39.73%, 40.53%, 41.16%, and 57.47%, respectively. A total of 2990 protein-coding genes, 16 rRNA genes (four 16S-23S-5S operons and one 16S-23S-5S-5S operon), 51 tRNA genes, 3 sRNAs, 214 interspersed repeated sequences, 106 tandem repeats, 80 minisatellite DNAs, 1 microsatellite DNAs, 8 genomic islands, 2 prophages, and 6 credible CRISPR loci were predicted in the chromosome. All the rRNA loci and predominant transcription of the protein-coding genes are in phase with the direction of DNA replication.

### Genome annotation

Total 1290 protein-coding genes in *L. brevis* CD0817 were assigned to 20 COG functional categories. The top four classes are: general function prediction only (190, 14.7%); translation, ribosomal structure and biogenesis (138, 10.7%); replication, recombination and repair (128, 9.9%); and amino acid transport and metabolism (127, 9.8%). The protein-coding genes involved in cell motility (2, 0.16%) represented the smallest group (Additional file 1: Fig. S1).

According to GO database, 1556 protein-coding genes belonging to three major categories of molecular function, cellular component and biological process were categorized into 35 subcategories (Additional file 1: Fig. S2). In the 9 subcategories of molecular function category, a majority of the genes were classified into catalytic and binding subcategories. Most genes were grouped into cell part and cell among the 7 subcategories of cellular component category. Within the 19 subcategories of biological process category, most genes were assigned to metabolic process and cellular process.

Altogether 1407 protein-coding genes were presumed to participate in 30 KEGG pathways (Additional file 1: Fig. S3). The largest category was membrane transport (275, 19.5%), followed by replication and repair (213, 15.1%), carbohydrate metabolism (199, 14.1%), and translation (174, 12.4%).

2563, 1030, and 2459 of the predicted 2990 proteins were classified into NR, Swiss-Prot, and TrEMBL functional categories, respectively.

### Comparative genomic analysis

**Fig. 2:**
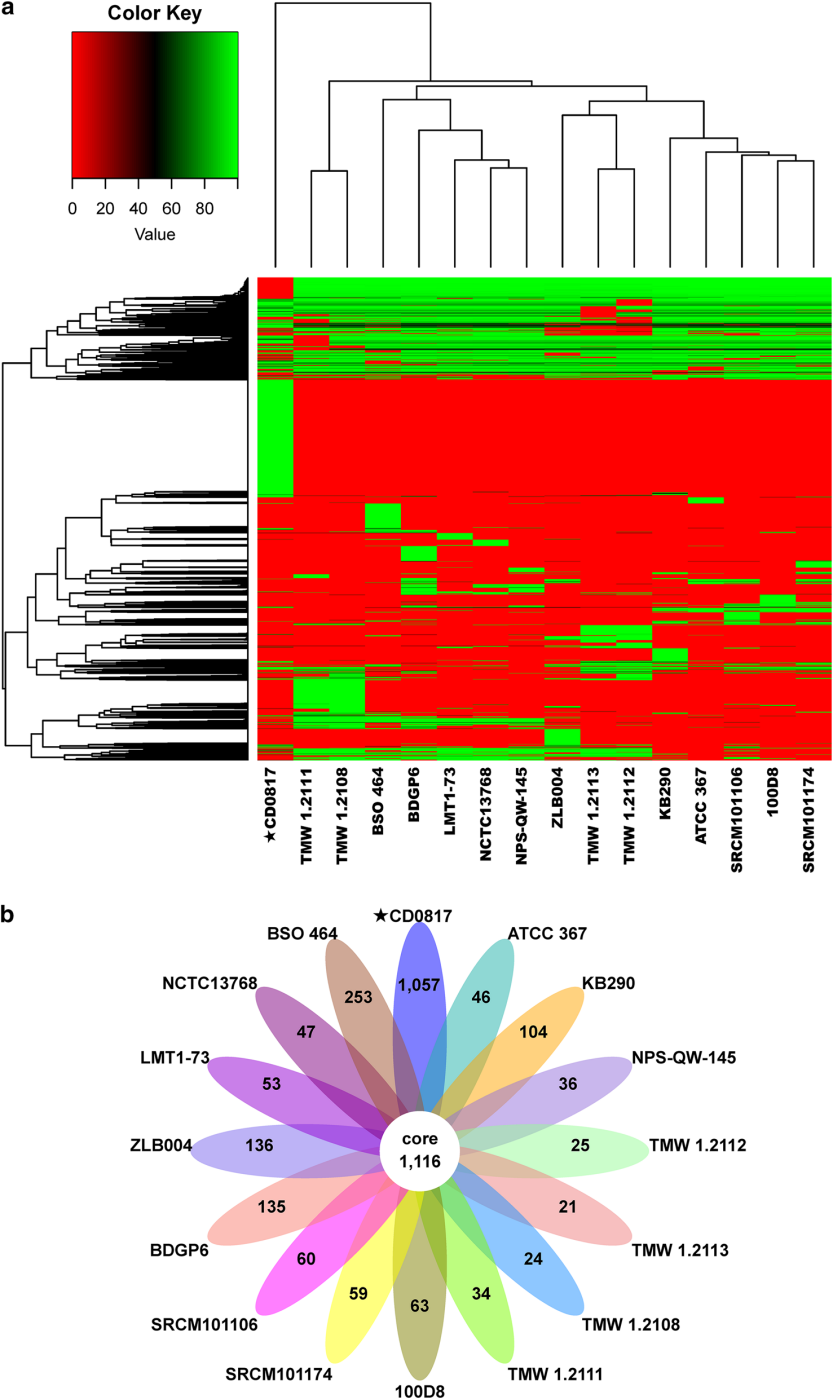
Core/Pan gene analysis of CD0817 and 15 completely sequenced reference *Lactobacillus brevis* strains. **a** Heatmap with the deletion of core gene. **b** Venn diagram of core and specific genes in each strain

To investigate the features that are present in CD0817, a comparative genomic analysis against 15 completely sequenced *L. brevis* strains was performed. Core/Pan gene analysis provided a core genome set of 1116 orthologs complemented by a dispensable genome set of 4250 genes, resulting in a pan-genome of 5366 genes. The number of core genes decreased while pan genes increased with the number of added strains (Additional file 1: Figs. S4 and S5). The heatmap after core gene deletion showed that CD0817 formed a distinct branch from the reference *L. brevis* strains, based on their gene contents (Fig. 2a). CD0817 had 1057 strain-specific genes (Fig. 2b).

**Fig. 3:**
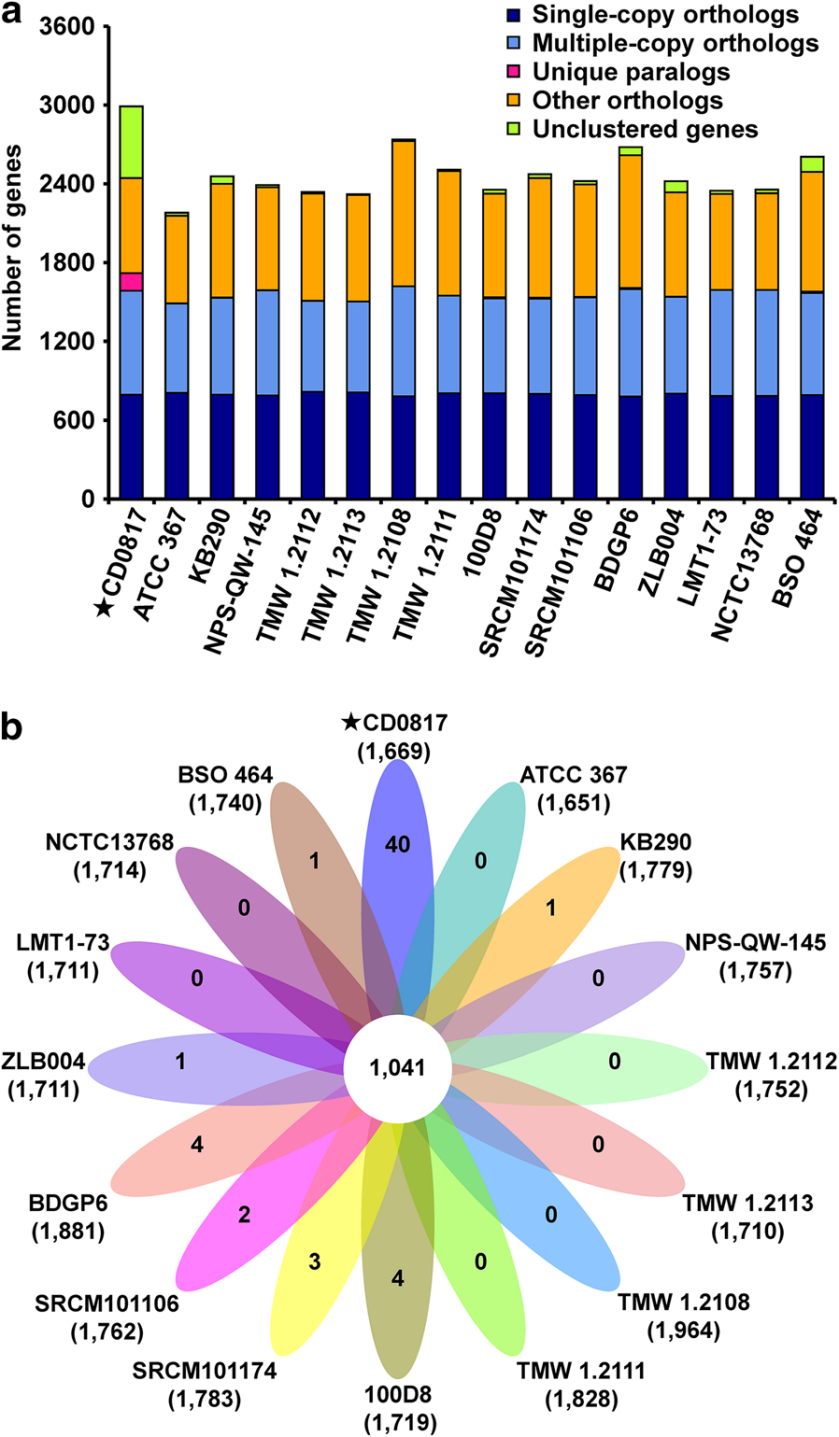
Gene family analysis of CD0817 and 15 completely sequenced reference *Lactobacillus brevis* strains. **a** Barchart of ortholog and paralog in each strain. **b** Venn diagram of shared and unique families in each strain. Number in the center of diagram indicates shared families. Numbers in outer end of ellipse indicate unique families. Numbers under strain names indicate total families

Gene family analysis revealed that total 2566 gene families were obtained among the 16 strains. Focusing on strain CD0817, 2444 genes were grouped into 1669 families with 40 of which were unique (Fig. 3).

**Fig. 4:**
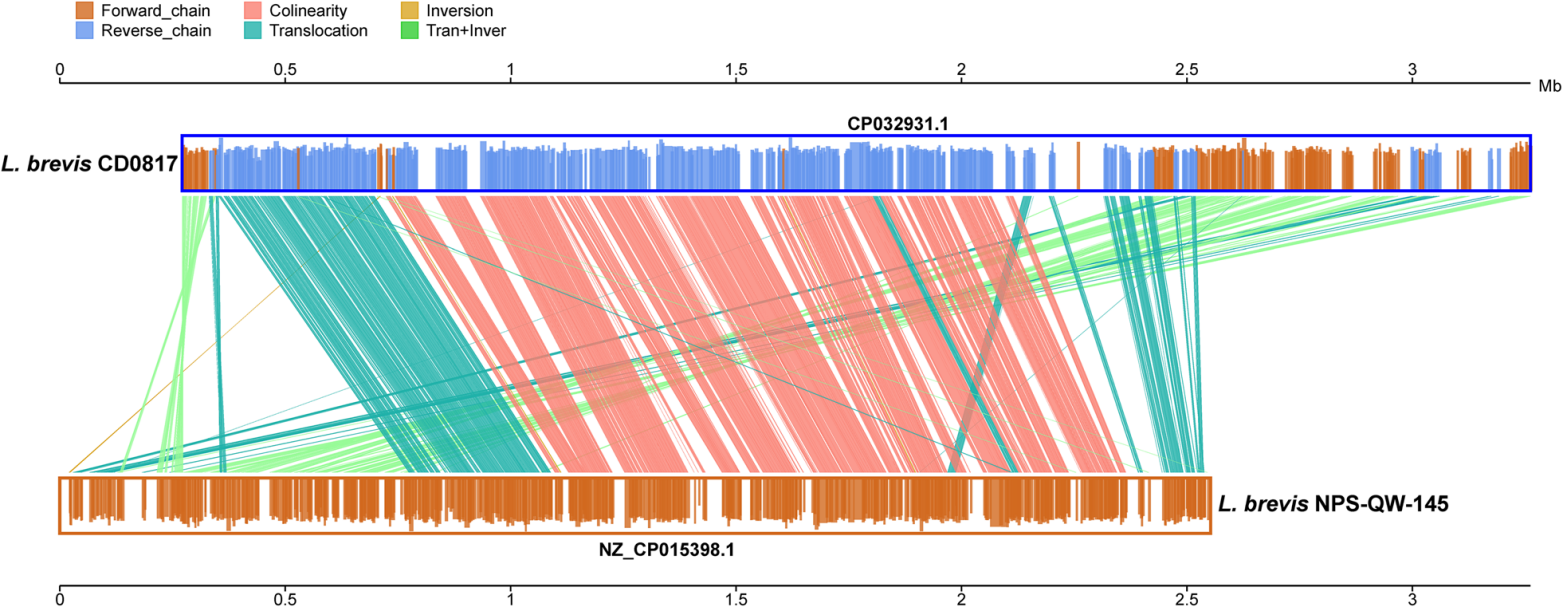
Synteny analysis between *L. brevis* strains CD0817 and NPS-QW-145

The gene synteny across the whole genomes of both *L. brevis* CD0817 and *L. brevis* NPS-QW-145 showed many gene translocation, inversion, and translocation plus inversion events occurred between these two strains (Fig. 4).

### Phylogenetic analysis

**Fig. 5:**
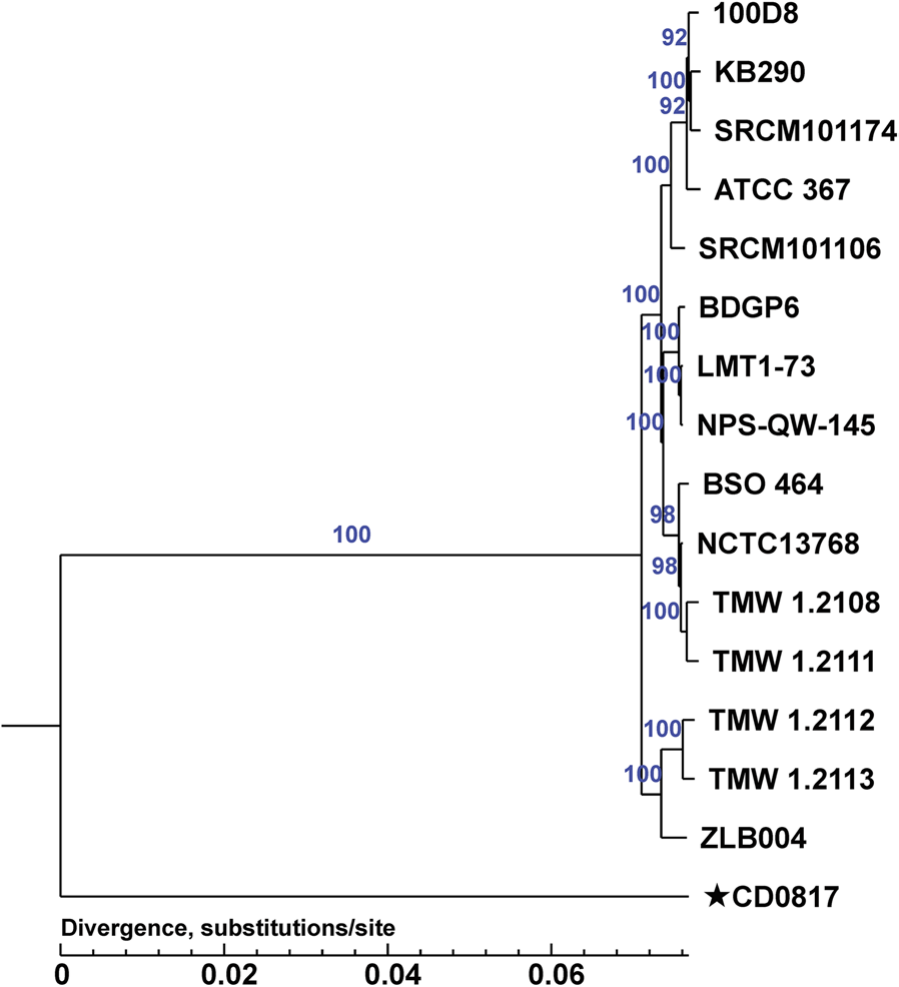
Neighbor-joining phylogenetic tree based on amino acid sequences of 718 single-copy orthologous genes detected from gene family analysis of CD0817 and 15 completely sequenced reference *Lactobacillus brevis* strains. Bootstrap values higher than 70% are shown at branch points

A neighbor-joining tree based on 718 single-copy orthologous genes detected from gene family analysis was constructed with 1000 replications in the bootstrap test. The phylogenetic tree shows that CD0817 diverged from other *L. brevis* strains in evolutionary process (Fig. 5).

### GAD genes

**Fig. 6:**
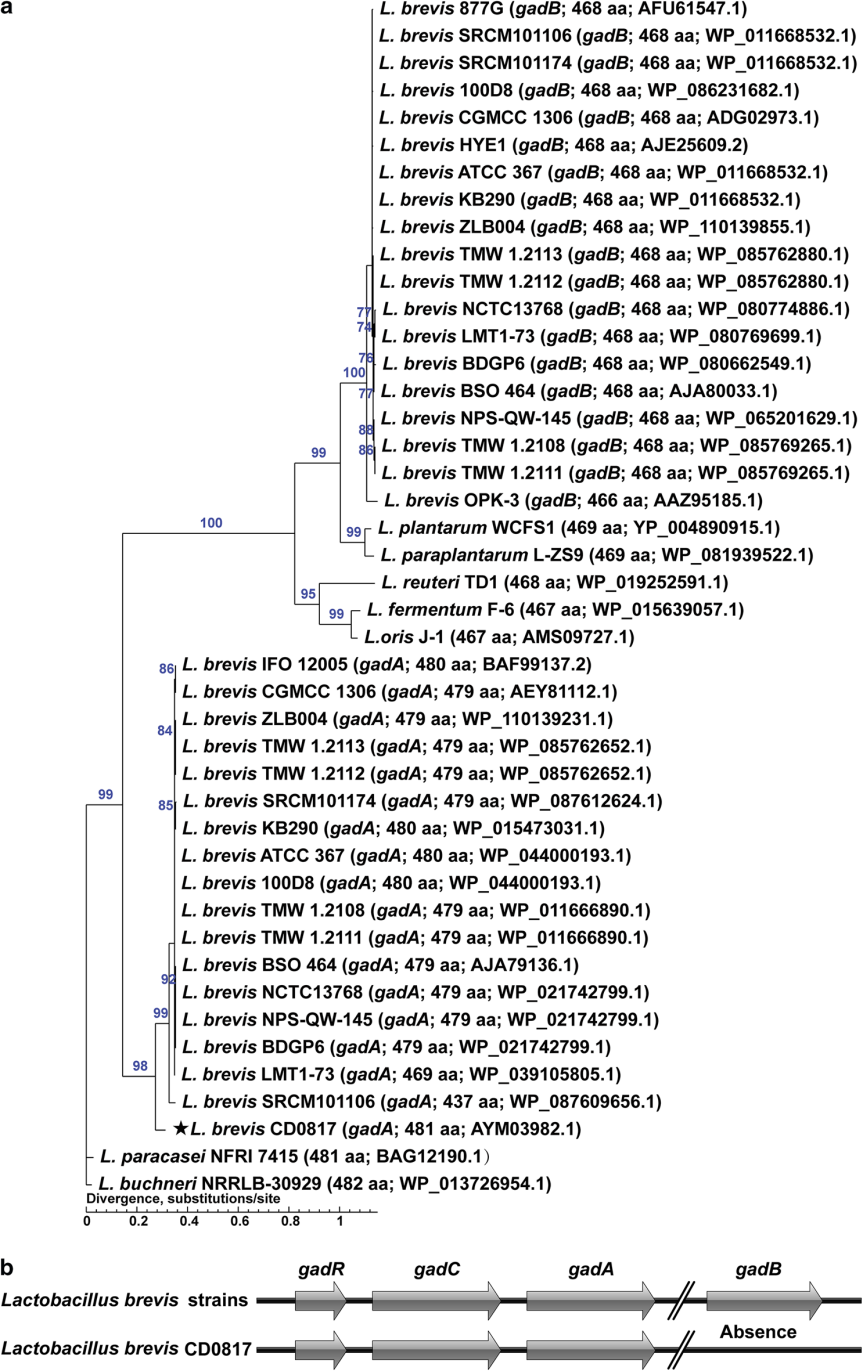
GAD analysis of CD0817 and 27 reference lactobacilli strains. **a** The maximum-likelihood tree based on amino acid sequences of GAD genes. Bootstrap values higher than 70% are shown at branch points. The length and GenBank accession numbers of GAD genes from each strain are shown in brackets. **b** Arrangements of GAD genes from CD0817 or other completely sequenced *Lactobacillus brevis* strains. *gadR*, transcriptional regulator gene; *gadC*, glutamate/GABA antiporter gene; *gadA*/*gadB*, GAD genes

To investigate lactobacilli GAD genes, a maximum-likelihood tree was constructed with 1000 replications in the bootstrap test (Fig. 6a). The phylogenetic tree shows that there are two GAD genes termed *gadA* (~ 479 aa) and *gadB* (~ 468 aa) in the low GABA-producing *L. brevis* genomes. However, *gadB* is absent from *L. brevis* CD0817 genome (Fig. 6b). Moreover, the GAD in CD0817 exhibits obvious difference from those in the other low GABA-producing *L. brevis* strains, as the amino acid sequence identity values of *gadA* and *gadC* in CD0817 against those in the other *L. brevis* strains are only 91% (Additional file 1: Fig. S6) and 90% (Additional file 1: Fig. S7), respectively.

## Discussion

Recently, we screened intestinal *L. brevis* CD0817, a strongest GABA-producing LAB strain (Chen et al. 2019). To facilitate elucidating its high yield molecular mechanism in the future research, we sequenced the genome of CD0817 in this work. CD0817 harbors a larger genome (3.10 Mb versus 2.34–2.92 Mb) with higher GC content (50.35% versus 45.27–46.04%) and more protein-coding genes (2990 versus 2180–2738) than the other completely sequenced *L. brevis* strains (Table 1).

COG is a database of proteins in which gene products are generally classified into dissimilar clusters of orthologous groups according to their homologous relationships (Tatusov et al. 1997). A total of 1290 *L. brevis* CD0817 protein-coding genes were assigned to 20 COG functional categories. The top four classes (general function prediction only; translation, ribosomal structure and biogenesis; replication, recombination and repair; and amino acid transport and metabolism) (Additional file 1: Fig. S1) were approximately consistent with those features in LAB (Barrangou et al. 2009; Makarova et al. 2006; Makarova and Koonin 2007).

Core genes were reduced while pan genes were increased with increasing strains (Additional file 1: Figs. S4 and S5), implying that the *L. brevis* strains analyzed harbor an open pan-genome (Li et al. 2014; Sun et al. 2015a). CD0817 possesses much more strain-specific genes than other *L. brevis* strains (1057 versus 21–253) (Fig. 2b). Whether these specific genes contribute to the high GABA production of CD0817 deserves further work. The whole genomic structures between *L. brevis* CD0817 and NPS-QW-145 were not very conserved, partially attributed to a lot of gene translocation, inversion, and translocation plus inversion events (Fig. 4).

The low GABA-producing *L. brevis* strains have an identical GAD system consisting of *gadCA* and *gadB* (Lyu et al. 2018; Shi and Li 2011; Wu et al. 2015; Zhang et al. 2010); however, these *L. brevis* strains showed various GABA-producing abilities. Clearly, the GAD system alone may be not sufficient to explicate the molecular basis for this difference in GABA production, implying that the generation of GABA may also be associated with complex cell physiology essentially ascribed to a genome (Lyu et al. 2017).

More interestingly, *L. brevis* CD0817 only containing *gadCA* (Fig. 6b) exhibited hitherto the most powerful lactic acid bacterial GABA production potential (Lyu et al. 2018; Wu and Shah 2015; Zhao et al. 2015). Although the exact molecular mechanism underlying the robust GABA formation ability by this “defective” GAD system in *L. brevis* CD0817 has yet to be elucidated, Lyu et al. (2018) recently verified in *L. brevis* CGMCC1306 that the *gadCA* operon was the major contributor to GABA production while the contribution of *gadB* was marginal, suggesting that *gadCA* operon rather than *gadB* was responsible for extracellular GABA accumulation (Wu et al. 2017). Therefore, the lack of *gadB* in CD0817 may not have a negative impact on the GABA synthesis. The extraordinary genome with a distinct GAD system (*gadCA*) may endow CD0817 with a unique cell physiological state conducive to GABA production.

In conclusion, the distinctive genome of a powerful GABA-producer *L. brevis* CD0817 was provided, followed by the comparative genomic analysis and discussion on this genome against 27 lactobacilli genomes. The generation of GABA may be related to not only GAD system but genome. This work may facilitate our understanding of the molecular mechanisms underlying the difference in lactic acid bacterial GABA-producing ability, thus enhancing GABA production by improving a LAB strain or metabolic regulation.

## Additional file

**Additional file 1: Fig. S1.** Distribution of protein-coding genes across COG functional categories in CD0817 genome. Note: one protein-coding gene might be assigned to more than one category. **Fig. S2.** Distribution of protein-coding genes across GO functional categories in CD0817 genome. Note: one protein-coding gene might be assigned to more than one category. **Fig. S3.** Distribution of protein-coding genes across KEGG pathway categories in CD0817 genome. Note: one protein-coding gene might be assigned to more than one category. **Fig. S4.** Dilution curve of core gene from CD0817 and 15 completely sequenced reference *Lactobacillus brevis* genomes. **Fig. S5.** Dilution curve of pan gene from CD0817 and 15 completely sequenced reference *Lactobacillus brevis* genomes. **Fig. S6.** Alignment of GadA primary structures of CD0817 and low GABA-producing lactobacilli strains. The amino acid residues in the black box are the conserved motif (HVDAA [S/F] GG); the amino acid in green box are the pyridoxal 5′-phosphate binding domain. **Fig. S7.** Alignment of GadC primary structures of CD0817 and low GABA-producing lactobacilli strains.

## Data Availability

The datasets supporting the conclusions of this article are included within the article.

## Abbreviations

*L. brevis*: *Lactobacillus brevis*
GABA: gamma-aminobutyric acid
LAB: lactic acid bacteria
GAD: glutamic acid decarboxylase
COG: clusters of orthologous groups
GO: gene ontology
KEGG: kyoto encyclopedia of genes and genomes
NR: non-redundant protein database
