# Supplementary material for: Genomic insights into a robust gamma-aminobutyric acid-producer Lactobacillus brevis CD0817
Source: AMB Express. 2019 May 24;9:72. doi: 10.1186/s13568-019-0799-0 (PMC6534642; doi:10.1186/s13568-019-0799-0)
Supplement: Supplementary file 1 — Additional file 1: Fig. S1. Distribution of protein-coding genes across COG functional categories in CD0817 genome. Note: one protein-coding gene might be assigned to more than one category. Fig. S2. Distribution of protein-coding genes across GO functional categories in CD0817 genome. Note: one protein-coding gene might be assigned to more than one category. Fig. S3. Distribution of protein-coding genes across KEGG pathway categories in CD0817 genome. Note: one protein-coding gene might be assigned to more than one category. Fig. S4. Dilution curve of core gene from CD0817 and 15 completely sequenced reference Lactobacillus brevis genomes. Fig. S5. Dilution curve of pan gene from CD0817 and 15 completely sequenced reference Lactobacillus brevis genomes. Fig. S6. Alignment of GadA primary structures of CD0817 and low GABA-producing lactobacilli strains. The amino acid residues in the black box are the conserved motif (HVDAA [S/F] GG); the amino acid in green box are the pyridoxal 5′-phosphate binding domain. Fig. S7. Alignment of GadC primary structures of CD0817 and low GABA-producing lactobacilli strains. [file 13568_2019_799_MOESM1_ESM.pdf]

Fig. S1 Distribution of protein-coding genes across COG functional categories in CD0817 genome. Note: one protein-coding gene might be assigned to more than one category

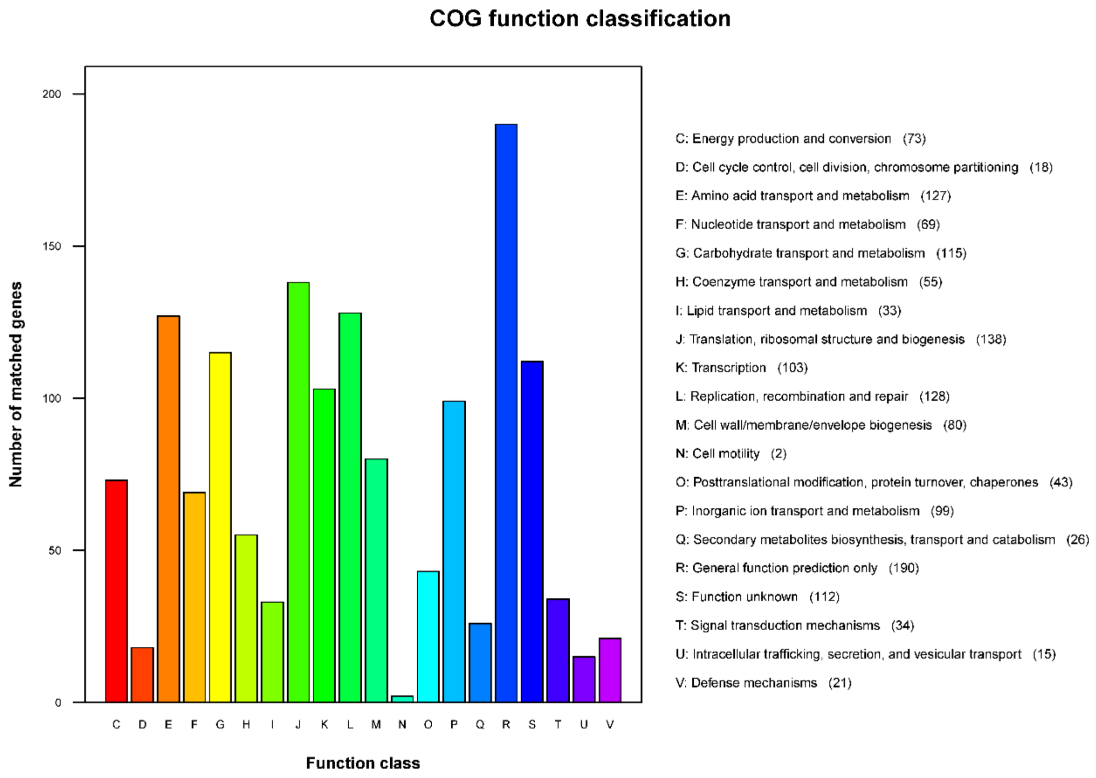

Fig. S2 Distribution of protein-coding genes across GO functional categories in CD0817 genome. Note: one protein-coding gene might be assigned to more than one category

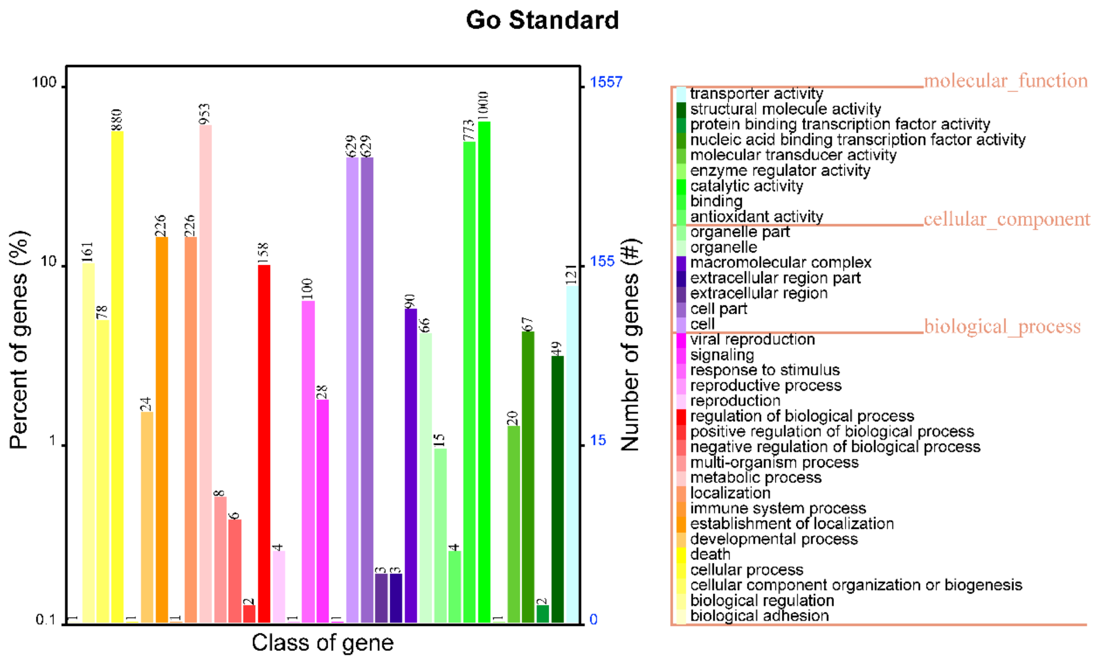

Fig. S3 Distribution of protein-coding genes across KEGG pathway categories in CD0817 genome. Note: one protein-coding gene might be assigned to more than one category

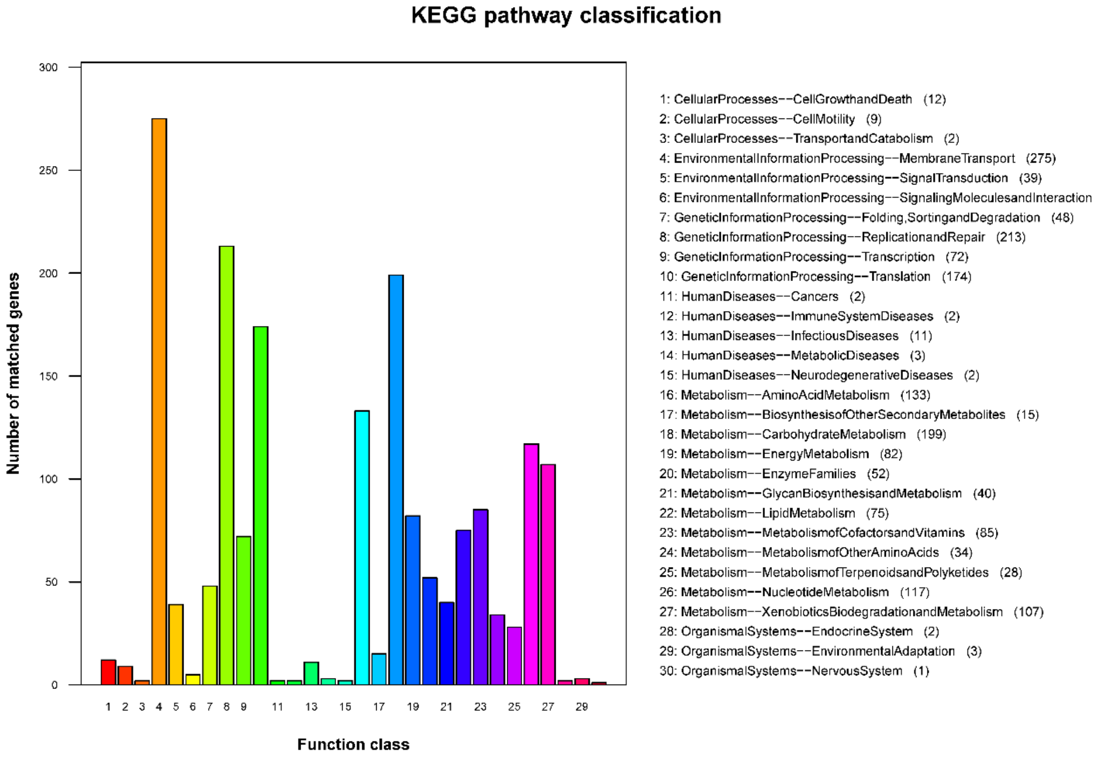

Fig. S4 Dilution curve of core genes from CD0817 and 15 completely sequenced reference *Lactobacillus brevis* genomes

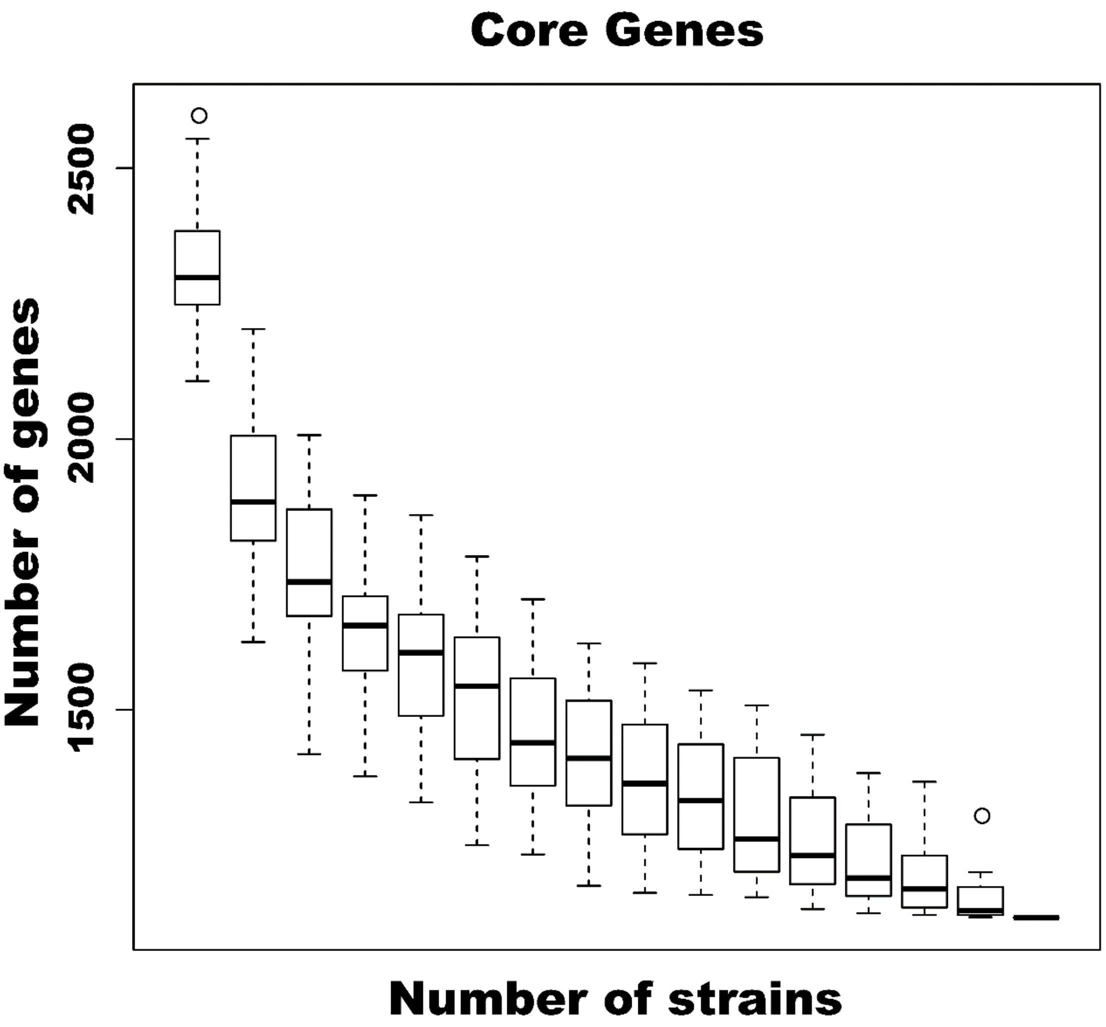

Fig. S5 Dilution curve of pan genes from CD0817 and 15 completely sequenced reference *Lactobacillus brevis* genomes

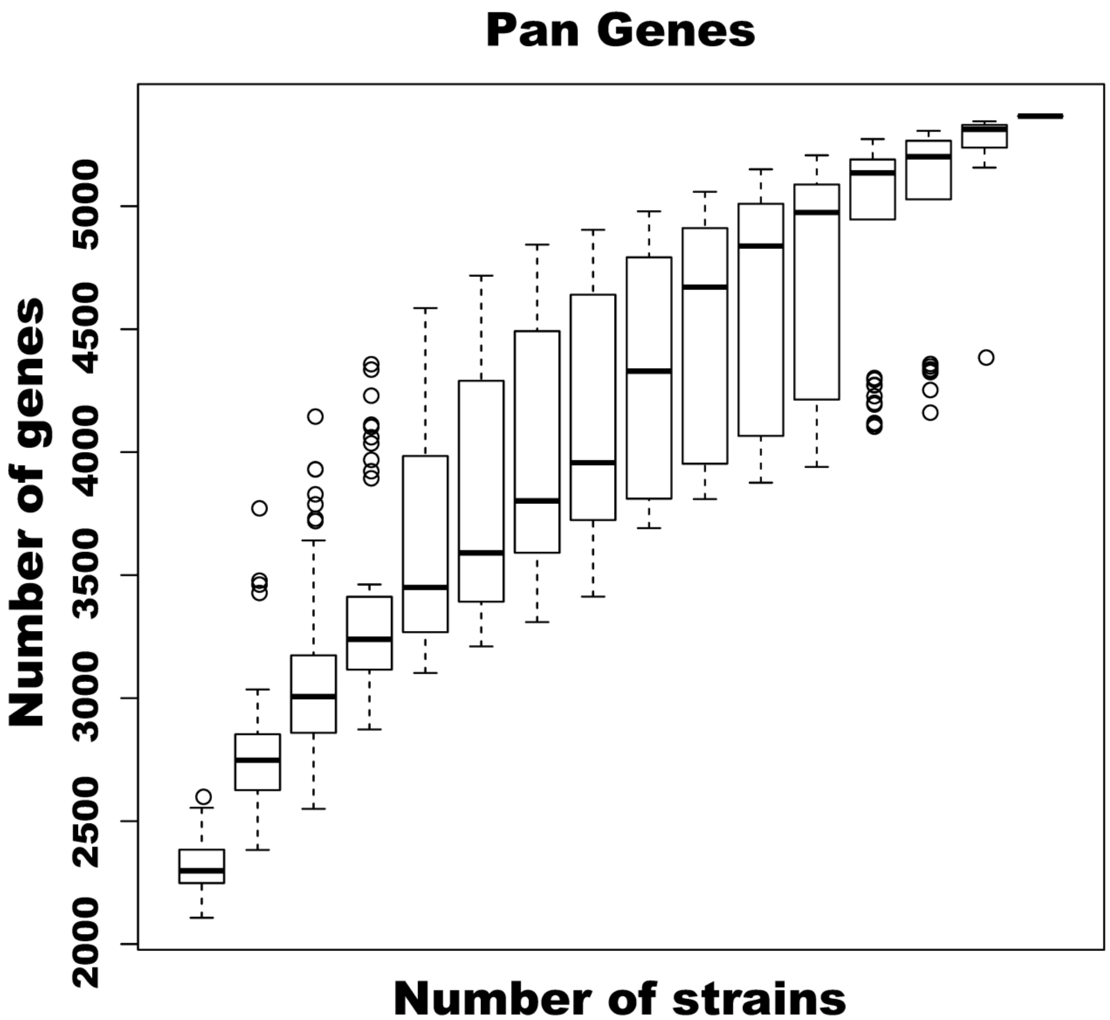

Fig. S6 Alignment of GadA primary structures of CD0817 and low GABA-producing lactobacilli strains. The amino acid residues in the black box are the conserved motif (HVDAA [S/F] GG); the amino acid in green box are the pyridoxal 5'-phosphate binding domain

```
L. brevis CD0817 gadA          .MTKQDQETQQIISDVDLEKTFLGSVEAGKSLPTNTLPEHPMAPDVAAQLVEHYRLNEAKA    60
L. brevis NPS-QW-145 gadA      .MNKNDQETQQMINNVDLEKTFLGSVEAGQSLPTNTLPDDPMAPDVAAQLVEHYRLNEAKA    60
L. brevis CGMCC 1306 gadA      .MNKNDQETQQMINNVDLEKTFLGSVEAGQSLPTNTLPDDPMAPDVAAQLVEHYRLNEAKA    60
L. brevis IFO 12005 gadA       MMNKNDQETQQMINNVDLEKTFLGSVEAGQSLPTYTLPDDPMAPDVAAQLVEHYRLNEAKA    61
L. paracasei NFRI 7415 gadA    ...MSEKNDEQMIDEIGLEQNFLGSVEAGKSLPTEELPEHPMPASIAAQLVQHHRLNEAKA    58
Consensus                                q i     le  flgsveag slpt  lp  pm    aaqlv h rlneaka

L. brevis CD0817 gadA          NQNLATFCTTQMEPQADELMKNALNTNAIDKSEYPKTAAMENYCVSMIAHLWGIPESEKIY   121
L. brevis NPS-QW-145 gadA      NQNLATFCTTQMEPQADELMKNALNTNAIDKSEYPKTAAMENYCVSMIAHLWGIPDNEKIY   121
L. brevis CGMCC 1306 gadA      NQNLATFCTTQMEPQADELMKNALNTNAIDKSEYPKTAAMENYCVSMIAHLWGIPDNEKIY   121
L. brevis IFO 12005 gadA       NQNLATFCTTQMEPQADELMKNALNTNAIDKSEYPKTAAMENYCVSMIAHLWGIPDNEKIY   122
L. paracasei NFRI 7415 gadA    NQNLATFCTTQMEPEADKLMTDALNTNAIDKSEYPKTAAMENYCVSMLAHLWGIPKGKKMY   119
Consensus                      nqnlatfcttqmep ad lm  alntnaidkseypktaamenycvsm ahlwgip   k y

L. brevis CD0817 gadA          DDFIGTSTVGSSEGCMLGGLALLHSWKHRAKDLGFDIDDLHTHKPNLVIMSGYQVVWEKFC   182
L. brevis NPS-QW-145 gadA      DDFIGTSTVGSSEGCMLGGLALLHSWKHRAKAAGFDIEDLHSHKPNLVIMSGYQVVWEKFC   182
L. brevis CGMCC 1306 gadA      DDFIGTSTVGSSEGCMLGGLALLHSWKHRAKAAGFDIEDLHSHKPNLVIMSGYQVVWEKFC   182
L. brevis IFO 12005 gadA       DDFIGTSTVGSSEGCMLGGLALLHSWKHRAKAAGFDIEDLHSHKPNLVIMSGYQVVWEKFC   183
L. paracasei NFRI 7415 gadA    KDFIGTSTVGSSEGCMLGGLSLLLSWKHRAEKAGFDTKDLHHHLPNLVIMSGYQVVWEKFC   180
Consensus                       dfigtstvgssegcmlggl ll swkhra   gfd  dlh h pnlvimsgyqvvwekfc

L. brevis CD0817 gadA          TYWNVEMRQVPINGDQVSLDMDSVMGYVDENTIGIIGIEGITYTGSVDDIQTLDQLVTEYN   243
L. brevis NPS-QW-145 gadA      TYWNVEMRQVPINGDQVSLDMDHVMDYVDENTIGIIGIEGITYTGSVDDIQTLDNLVSEYN   243
L. brevis CGMCC 1306 gadA      TYWNVEMRQVPINGDQVSLDMDHVMDYVDENTIGIIGIEGITYTGSVDDIQTLDNLVTEYN   243
L. brevis IFO 12005 gadA       TYWNVEMRQVPINGDQVSLDMDHVMDYVDENTIGIIGIEGITYTGSVDDIQTLDNLVTEYN   244
L. paracasei NFRI 7415 gadA    TYWNVELRQVPIDQNHMSMDMDHVMDYVDENTIGIVGIQGITYTGAVDDIQKLDRLVSEYN   241
Consensus                      tywnve rqvpi     s dmd vm yvdentigi gi gitytg vddiq ld lv eyn

L. brevis CD0817 gadA          KTAVMEVHIHVDAAFGGLFAPFVDGFKPWDFRLKNVVSINVSGHKYGMVYPGLGWIVWRHN   304
L. brevis NPS-QW-145 gadA      KTATMEVRIHVDAAFGGLFAPFVDGFNPWDFRLKNVVSINVSGHKYGMVYPGLGWIVWRHN   304
L. brevis CGMCC 1306 gadA      KTATMEVRIHVDAAFGGLFAPFVDGFNPWDFRLKNVVSINVSGHKYGMVYPGLGWIVWRHD   304
L. brevis IFO 12005 gadA       KTATMEVRIHVDAAFGGLFAPFVDGFNPWDFRLKNVVSINVSGHKYGMVYPGLGWIVWRHD   305
L. paracasei NFRI 7415 gadA    KTAVLEIRIHVDSAFGGLFAPFVDGFKPWDFRLKNVVSINVSGHKYGMVYPGIGWIVWRNN   302
Consensus                      kta  p  ihvd afgglfapfvdgf pwdfrlknvvsinvsghkygmvypg gwivwr

L. brevis CD0817 gadA          NADTLPEEMRFQVPYLGKTVDSIAINFSHSGAHISAQYYNFIRFGKDGYKAIMQNVRKVSL   365
L. brevis NPS-QW-145 gadA      TADILPAEMRFQVPYLGKTVDSIAINFSHSGAHISAQYYNFIRFGLSGYKTIMQNVRKVSL   365
L. brevis CGMCC 1306 gadA      TADILPAEMRFQVPYLGKTVDSIAINFSHSGAHISAQYYNFIRFGLSGYKTIMQNVRKVSL   365
L. brevis IFO 12005 gadA       TADILPAEMRFQVPYLGKTVDSIAINFSHSGAHISAQYYNFIRFGLSGYKTIMQNVRKVSL   366
L. paracasei NFRI 7415 gadA    SEDLLPKEMRFSVPYLGSSVDSIAINFSHSGAHIVGQYHNFVRFGYKGYEAIMNNVRKVSL   363
Consensus                        d lp emrf vpylg  vdsiainfshsgahi  qy nf rfg  gy  im nvrkvsl

L. brevis CD0817 gadA          KLTDALKKFGIFDILVDGSQLPINCWKLADDANVKWTLYDLESELAKYGWQVPAYPLPKNR   426
L. brevis NPS-QW-145 gadA      KLTAALKTYGIFDILVDGSQLPINCWKLADDAPVGWTLYDLESELAKYGWQVPAYPLPKNR   426
L. brevis CGMCC 1306 gadA      KLTAALKTYGIFDILVDGSQLPINCWKLADDAPVGWTLYDLESELAKYGWQVPAYPLPKNR   426
L. brevis IFO 12005 gadA       KLTAALKTYGIFDILVDGSQLPINCWKLADDAPVGWTLYDLESELAKYGWQVPAYPLPKNR   427
L. paracasei NFRI 7415 gadA    RITEELKKFGIFEILNDGSQLPINCWKLADDAKVDWTLYDLEGELAKYGWQVPAYPLPKNR   424
Consensus                        t  lk  gif il dgsqlpincwkladda v wtlydle elakygwqvpayplpknr

L. brevis CD0817 gadA          DDVTISRIVVRPSMTMTIADDFLDDLKLAIDGLNHSFGTE..DAANADTDNKTTVH   480
L. brevis NPS-QW-145 gadA      DDVTISRIVVRPSMTMTIADDFLDDLKLAIDGLNHTFG....VTTTVDQDNKTTVR   478
L. brevis CGMCC 1306 gadA      DDVTISRIVVRPSMTMTIADDFLDDLKLAIDGLNHTFG....VTTTVDQDNKTTVR   478
L. brevis IFO 12005 gadA       DDVTISRIVVRPSMTMTIADDFLDDLKLAIDGLNHTFG....VTTTVDQDNKTTVR   479
L. paracasei NFRI 7415 gadA    EDTTISRIVVRPSMTMTILDDFMEDLKMAIHNLNKEHGNNELEYNIPSAADATTVS   480
Consensus                       d tisrivvrpsmtmti ddf  dlk ai  ln   g              ttv
```

Fig. S7 Alignment of GadC primary structures of CD0817 and low GABA-producing lactobacilli strains.

```
L. brevis CD0817 gadC        MDENKSGQQIDTQKANRITGFQLFSMTTSMVMTVYGFAAFAKQGPTAFFYLFLAGILWFLPVT     63
L. brevis NPS-QW-145 gadC    MDENKSEQQIDTQKANRITGFQLFSMTTSMVMTVYGFAAFAKQGPTAFFYLFLAGILWFLPVT     63
L. brevis CGMCC 1306 gadC    MDENKSEQQIDTQKANRITGFQLFSMTTSMVMTVYGFAAFAKQGPTAFFYLFLAGILWFLPVT     63
L. brevis IFO-12005 gadC     .........................................................MVFTGT      6
Consensus                                                                                  t

L. brevis CD0817 gadC        RTSGEMASIDGWSKGGIFTWSRNMLGERAGWSALFYQWIHITVGMNTMMYFIIGCFSVTFGLP    126
L. brevis NPS-QW-145 gadC    RTSGEMASIDGWSKGGIFTWSRNMLGERAGWSALFYQWIHITVGMNTMMYFIIGCFSVTFGLP    126
L. brevis CGMCC 1306 gadC    RTSGEMASIDGWSKGGIFTWSRNMLGERAGWSALFYQWIHITVGMNTMMYFIIGCFSVTFGLP    126
L. brevis IFO-12005 gadC     RTSGEMASIDGWSKGGIFTWSRNMLGERAGWSALFYQWIHITVGMNTMMYFIIGCFSVTFGLP     69
Consensus                    rtsgemasidgwskggiftwsrnmlgeragwsalfyqwihitvgmntmmyfiigcfsvtfglp

L. brevis CD0817 gadC        IITDNPLVKFILMMVILWGLIALQQRGTSVTGKIAQWCFTLGVIIPVFFLLFLFIMYLVKGNP    189
L. brevis NPS-QW-145 gadC    IITDNPLVKFILMMIILWGLIFLQQKGTSVTGKIAQWCFTLGVIIPVFFLLFLFILYLVQGNP    189
L. brevis CGMCC 1306 gadC    IITDNPLVKFILMMIILWGLIFLQQKGTSVTGKIAQWCFTLGVIIPVFFLLFLFILYLVQGNP    189
L. brevis IFO-12005 gadC     IITDNPLVKFILMMIILWGLIFLQQKGTSVTGKIAQWCFTLGVIIPVFFLLFLFILYLVQGNP    132
Consensus                    iitdnplvkfilmm ilwgli lqq gtsvtgkiaqwcftlgviipvffllflfi ylv gnp

L. brevis CD0817 gadC        TMIDVNVHTILPKNWSGSVLVGFVPFILAFAGAEGSAPHVKDLDKPSVYPKVMMALAIAAICS    252
L. brevis NPS-QW-145 gadC    AMIHVNMHTIFPSSWNGSVLVGFVPFILAFAGAEGSAPHVKDLDKPSIYPKVMMALAVAAICS    252
L. brevis CGMCC 1306 gadC    AMIHVNMHTIFPSSWNGSVLVGFVPFILAFAGAEGSAPHVKDLDKPSIYPKVMMALAVAAICS    252
L. brevis IFO-12005 gadC     AMIHVNMHTIFPSSWNGSVLVGFVPFILAFAGAEGSAPHVKDLDKPSIYPKVMMALAVAAICS    195
Consensus                     mi vn hti p  w gsvlvgfvpfilafagaegsaphvkdldkps ypkvmmala aaics

L. brevis CD0817 gadC        DIIGSMAIAMTIPNDQIQLSNGIVYAYGALVKDYGVSVVFVEKLTGFLLAVGVLGEISSWVVG    315
L. brevis NPS-QW-145 gadC    DIIGSMAIAMTIPNNQIQLSNGIVYAYGALVARYGVGVVFVEKLTGFLLAVGVLGEISSWVVG    315
L. brevis CGMCC 1306 gadC    DIIGSMAIAMTIPNNQIQLSNGIVYAYGALVARYGVGVVFVEKLTGFLLAVGVLGEISSWVVG    315
L. brevis IFO-12005 gadC     DIIGSMAIAMTIPNNQIQLSNGIVYAYGALVARYGVGVVFVEKLTGFLLAVGVLGEISSWVVG    258
Consensus                    diigsmaiamtipn qiqlsngivyaygalv  ygv vvfvekltgfllavgvlgeisswvvg

L. brevis CD0817 gadC        PNSGMFEAAKAGYLPPRFSKANKYGIETNVMILQGVIVSIVGALLTFGAGGNKSSLSFQIAMS    378
L. brevis NPS-QW-145 gadC    PNAGMFEAAKAGYLPPRFSKANKYGIETNVMVLQGVIVSIVGALLTFGAGGNKASLSFQTAMS    378
L. brevis CGMCC 1306 gadC    PNAGMFEAAKAGYLPPRFSKANKYGIETNVMVLQGVIVSIVGALLTFGAGGNKASLSFQTAMS    378
L. brevis IFO-12005 gadC     PNAGMFEAAKAGYLPPRFSKANKYGIETNVMVLQGVIVSIVGALLTFGAGGNKASLSFQTAMS    321
Consensus                    pn gmfeaakagylpprfskankygietnvm lqgvivsivgalltfgaggnk slsfq ams

L. brevis CD0817 gadC        LTVALYTLMYMLMFISYLVLQFRYKDLHRSFVASKSSTLRIVYGGLGFILSAFGFVVTFFPPA    441
L. brevis NPS-QW-145 gadC    LTVALYTLMYMLMFISYLVLQFKYVDLHRDFVAAKSRWLRITYGILGFILSAFGFVVTFFPPA    441
L. brevis CGMCC 1306 gadC    LTVALYTLMYMLMFISYLVLQFKYVDLHRDFVAAKSRWLRITYGILGFILSAFGFVVTFFPPA    441
L. brevis IFO-12005 gadC     LTVALYTLMYMLMFISYLVLQFKYVDLHRDFVAAKSRWLRITYGILGFILSAFGFVVTFFPPA    384
Consensus                    ltvalytlmymlmfisylvlqf y dlhr fva ks  lri yg lgfilsafgfvvtffppa

L. brevis CD0817 gadC        DLSLASKHTYSILLISAFVVMLVLPFILYRFHERWANQLGINVDEVPGVAADESLEKET        500
L. brevis NPS-QW-145 gadC    DLSVASKHTYLMLLVSAFVVMLVLPFILYRFHDKWALQLGVNVDEVAAPTGHEALEKET        500
L. brevis CGMCC 1306 gadC    DLSVASKHTYLMLLVSAFVVMLVLPFILYRFHDKWALQLGVNVDEVAAPTGHEALEKET        500
L. brevis IFO-12005 gadC     DLSVASKHTYLMLLVSAFVVMLVLPFILYRFHDKWALQLGVNVDEVAAPTGHEALEKET        443
Consensus                    dls askhty  ll safvvmlvlpfilyrfh  wa qlg nvdev      e leket
```
